# Supplementary material for: Metabonomics and Transcriptomic Analysis of Free Fatty Acid Synthesis in Seedless and Tenera Oil Palm
Source: Int J Mol Sci. 2024 Jan 30;25(3):1686. doi: 10.3390/ijms25031686 (PMC10855455; doi:10.3390/ijms25031686)
Supplement: Supplementary file 1 [file ijms-25-01686-s001.zip › ijms-2822011-supplementary.pdf]

Supplementary material

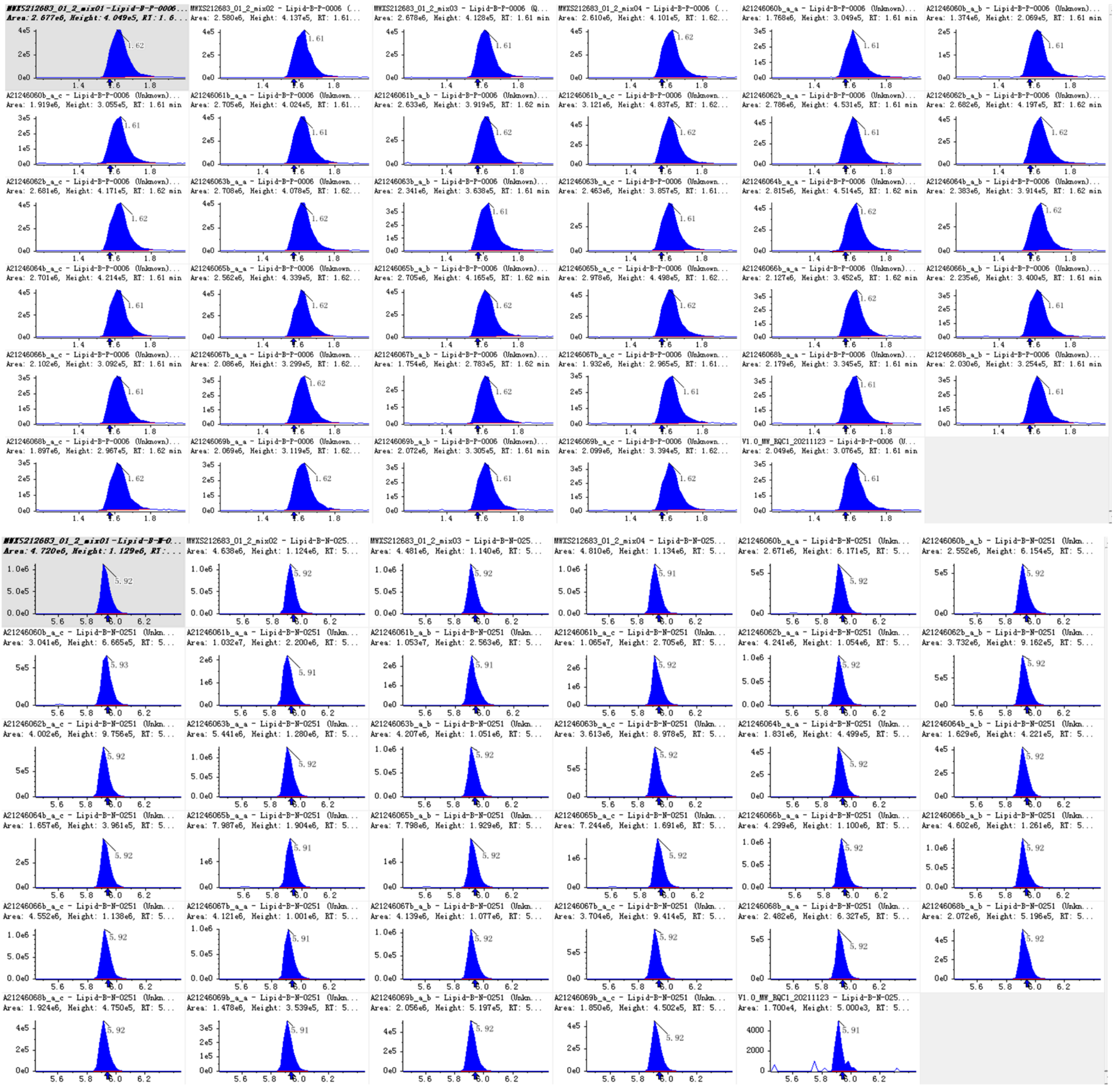

Supplementary Figure S1:Correction chart for mass spectrometry integration of fatty acid metabolites

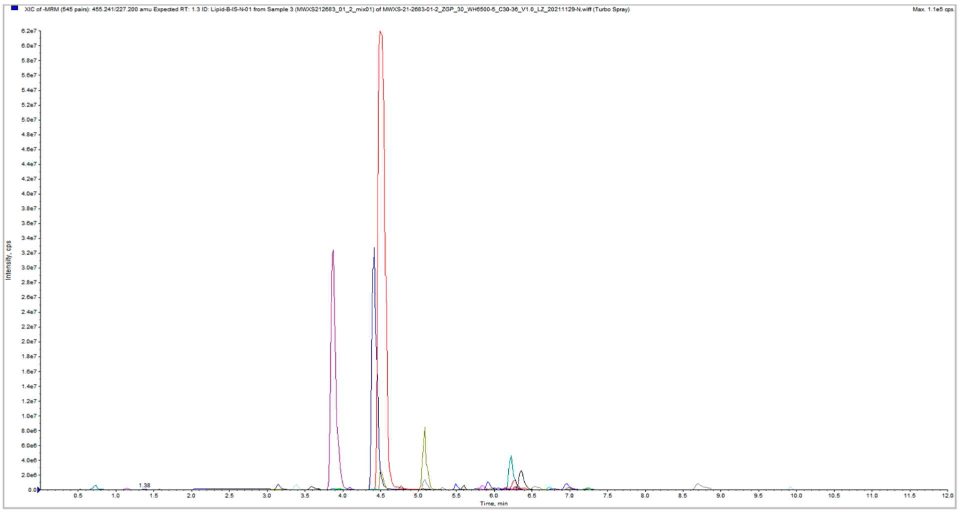

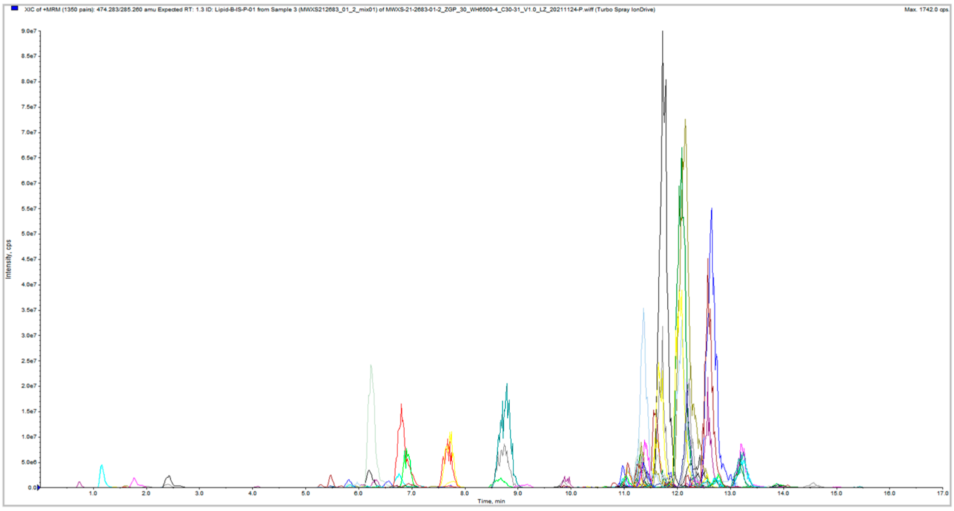

XIC of +MRM (1350 pairs): 474.283/285.260 amu Expected RT: 1.3 ID: Lipid-B-IS-P-01 from Sample 3 (MWXS212683_01_2_mix01) of MWXS-21-2683-01-2_ZGP_30_WH6500-4_C30-31_V1.0_LZ_20211124-P.wiff (Turbo Spray IonDrive)
Max. 1742.0 cps.
Intensity, cps
Time, min

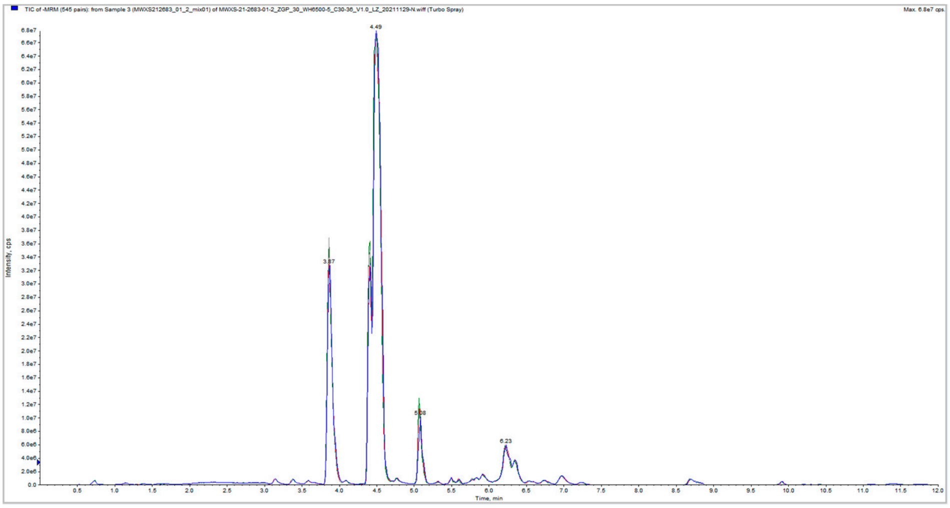

TIC of -MRM (545 pairs): from Sample 3 (MWXS212683_01_2_mix01) of MWXS-21-2683-01-2_ZGP_30_WH6500-5_C30-36_V1.0_LZ_20211129-N.wiff (Turbo Spray)
Max. 6.8e7 cps.
4.49
3.87
5.08
6.23
Intensity, cps
Time, min

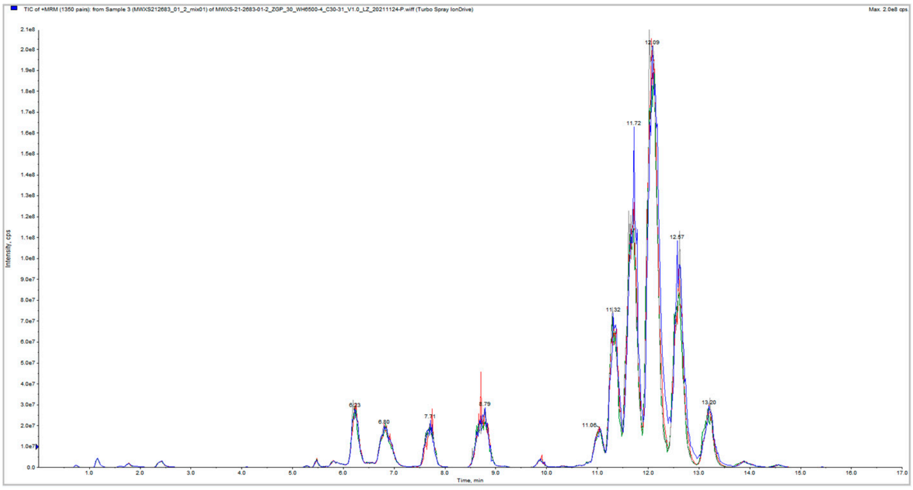

TIC of +MRM (1350 pairs): from Sample 3 (MWXS212683_01_2_mix01) of MWXS-21-2683-01-2_ZGP_30_WH6500-4_C30-31_V1.0_LZ_20211124-P.wiff (Turbo Spray IonDrive)
Max. 2.0e8 cps.
12.09
11.72
12.57
11.32
6.23
6.80
7.71
8.79
11.06
13.20
Intensity, cps
Time, min

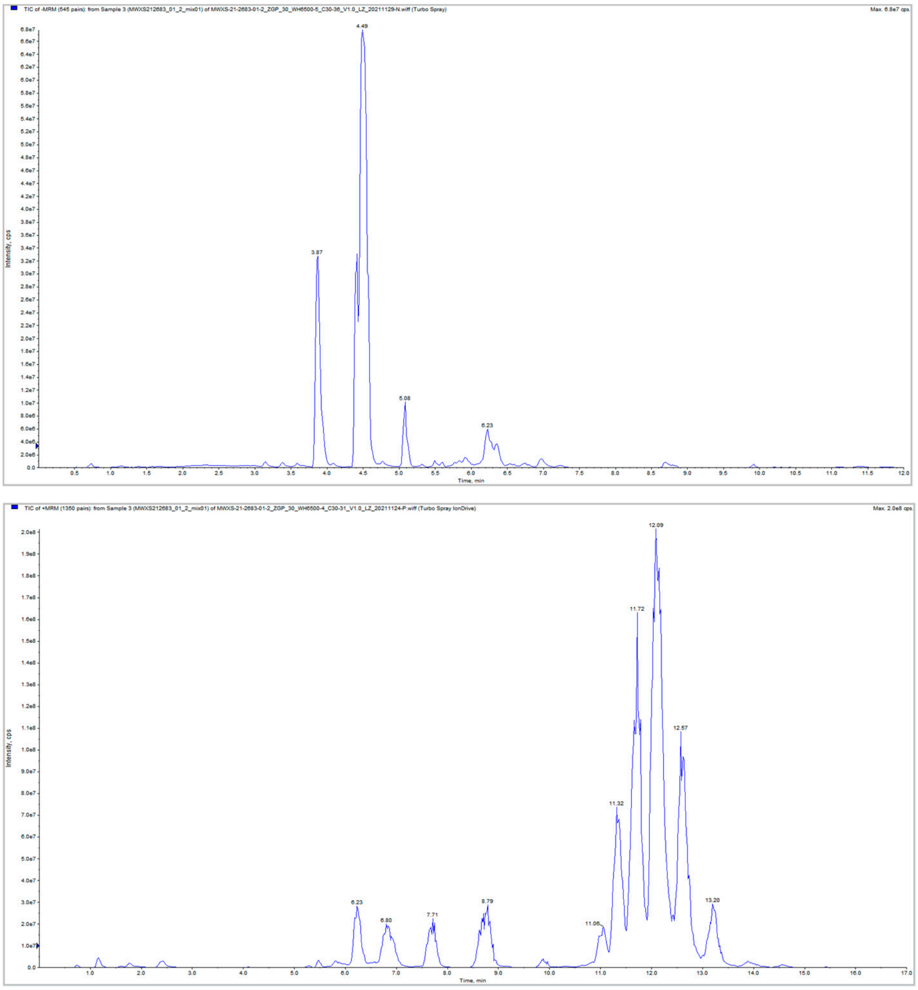

Supplementary Figure S2:The MRM model

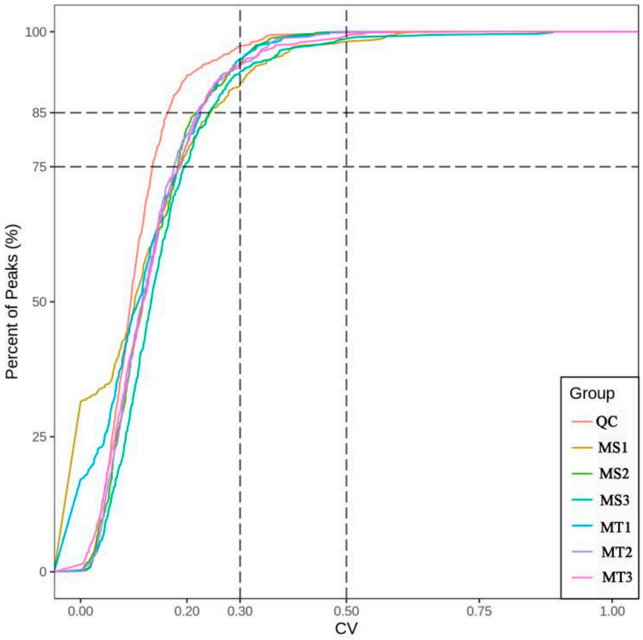

Supplementary Figure S3:CV distribution map in samples MS1, MS2, MS3, MT1, MT2, and MT3

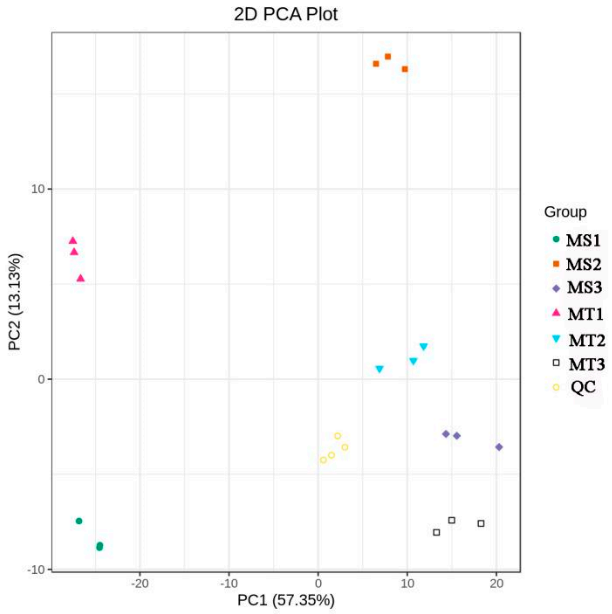

Supplementary Figure S4:Component analysis of MS and MT overall samples.QC is the quality control sample.

Supplementary Table S1: Mass of RNA-seq

Sample	Raw Reads	Clean Reads	Clean Base(G)	Error Rate(%)	Q20(%)	Q30(%)	GC Content(%)
MS1-1	48208652	45791886	6.87	0.03	97.46	92.88	49.12
MS1-2	48099228	45286668	6.79	0.03	97.66	93.28	47.81
MS1-3	47892422	44799750	6.72	0.03	97.67	93.34	49.07
MS2-1	48211024	45808550	6.87	0.03	97.67	93.34	48.73
MS2-2	46263496	44045448	6.61	0.03	97.50	92.91	47.86
MS2-3	45421048	42279992	6.34	0.03	97.77	93.48	47.66
MS3-1	48740904	46390726	6.96	0.03	97.76	93.52	48.97
MS3-2	47079366	45077920	6.76	0.03	97.65	93.29	48.99
MS3-3	57890096	56348144	8.45	0.03	97.39	92.85	48.47
MT1-1	46359398	43300400	6.50	0.03	97.73	93.42	47.67
MT1-2	45890134	42273062	6.34	0.03	97.67	93.33	47.82
MT1-3	47574154	44909076	6.74	0.03	97.51	92.98	48.31
MT2-1	48992046	45486604	6.82	0.03	97.72	93.46	48.81
MT2-2	46921290	43943568	6.59	0.03	97.64	93.26	48.44
MT2-3	47917708	44238738	6.64	0.03	97.58	93.10	48.65
MT3-1	48145634	44847734	6.73	0.03	97.67	93.32	48.73
MT3-2	46633640	43299316	6.49	0.03	97.18	92.16	48.83
MT3-3	47406742	43203442	6.48	0.03	97.86	93.95	48.56

Supplementary Table S2:Design of primer for qRT-PCR

**Primer**	**Sequence (5 '-3')**
BC-qPCR-F	CCAGGTTCAGGAGGTTGCTG

BC-qPCR-R	CTGGTGCTGCAGTTTGGCTA
SAD-qPCR-F	GGGAGGTCTCGGTGTAAGGA
SAD-qPCR-R	ACAAACACCAGCAGAGACCC
FATA-qPCR-F	CAGGAGAGCCGATTTGGACA
FATA-qPCR-R	TGCTTTCAAGGACCCATCCG
FabI-qPCR-F	GTTCTCAAATGCCCGGCTTC
FabI-qPCR-R	GGGCTTTTGGAATCCTGTTCA
ACC-qPCR-F	GCTGATCCTGAGGTGAAGCA
ACC-qPCR-R	TTCCTTGGCCCTTGCAATCT
FabB-qPCR-F	CGACACCGTCCCCAATGTAA
FabB-qPCR-R	TTGTGACCCCCGAAACCAAA
FabG4-qPCR-F	TACCATTGATGGCGGGATGG
FabG4-qPCR-R	AAGCCGGAGCACATTACCAA
FabZ-qPCR-F	TCTTTGCCGCTTCTGTCTGT
FabZ-qPCR-F	AGCAAGGAAGACTCTGCCAA
FatB-qPCR-R	CAGCCAAGGGTTCCTGATCT
FatB-qPCR-F	TTCGACGCTTTTGCTGATGC
FAD6-qPCR-R	AGCGGGGTGGGATTTTAGTG
FAD6-qPCR-F	ACAGAGGAACCGCAGAGAGA
